# Large-scale controlled fabrication of highly roughened flower-like silver nanostructures in liquid crystalline phase

**DOI:** 10.1038/srep12355

**Published:** 2015-07-28

**Authors:** Chengliang Yang, Xiangjun Xiang, Ying Zhang, Zenghui Peng, Zhaoliang Cao, Junlin Wang, Li Xuan

**Affiliations:** 1 State Key Laboratory of Applied Optics, Changchun Institute of Optics, Fine Mechanics and Physics, Chinese Academy of Sciences, Changchun, Jilin 130033, China; 2 University of the Chinese Academy of Sciences, Beijing 100039, China

## Abstract

Large-scale controllable fabrication of highly roughened flower-like silver nanostructures is demonstrated experimentally via electrodeposition in the liquid crystalline phase. Different sizes of silver flowers are fabricated by adjusting the deposition time and the concentration of the silver nitrate solution. The density of the silver flowers in the sample is also controllable in this work. The flower-like silver nanostructures can serve as effective surface-enhanced Raman scattering and surface-enhanced fluorescence substrates because of their local surface plasmon resonance, and they may have applications in photoluminescence and catalysis. This liquid crystalline phase is used as a soft template for fabricating flower-like silver nanostructures for the first time, and this approach is suitable for large-scale uniform fabrication up to several centimetres.

Noble metal nanostructures have attracted extensive attention due to their special physical and chemical properties^1^,^2^,^3^,^4^,^5^ and their potential applications in catalysis and photonics, such as surface-enhanced Raman scattering (SERS) and surface-enhanced fluorescence (SEF)^6^,^7^,^8^,^9^,^10^,^11^,^12^,^13^,^14^. Among various specific metal nanostructures, flower-like silver nanostructures arouse more interest because of their high degree of SERS and SEF enhancement effects and their chemical stability. In these applications, the nanostructures’ size, morphology and uniformity affect the localized surface-plasmon resonance^15^,^16^ strongly. Therefore, controllable fabrication is one of the most important aspects on which to focus an experiment. In recent decades, there have been many reports about such structures and their formation from both experimental and theoretical perspectives^17^,^18^,^19^,^20^,^21^. Several fabrication methods have been reported, including electron beam lithography, nanosphere lithography, seed-mediated growth, pH-dependent self-assembly and various chemical aggregations^22^,^23^,^24^,^25^. Lithography is a complicated top-down method for nanoscale fabrication that is very precise in creating geometric patterns but is extremely slow and time-consuming. In contrast, self-assembly and aggregation are typical bottom-up techniques that are usually quite fast, simple and inexpensive. The shortcomings of bottom-up processes are also obvious, such as difficulties in controlling the product’s size and morphology and in performing large-scale uniform growth.

In this study, a simple method is demonstrated for the large-scale controllable fabrication of flower-like silver nanostructures. The fabrication method is electrodeposition in ternary liquid crystalline phases^26^. Different sizes of flower-like silver nanostructures, ranging from 250 nm to 1.5 μm and different coverage ratios of the samples are obtained, which can be controlled by adjusting the deposition time and the concentration of the silver nitrate solution. Large-scale uniform fabrication for several centimetres also can be performed by using this approach, which makes it very suitable for the production of surface-enhanced Raman scattering and surface-enhanced fluorescence substrates.

## Results

### Fabrication

In this work, we use the liquid crystalline phase as a soft template to perform the electrodeposition process. The liquid crystalline phase is prepared according to the ternary phase diagram, which consists of the anionic surfactant sodium bis(2-ethylhexyl) sulfosuccinate (AOT), the oil phase p-xylene and water. The electrodeposition process is prepared and carried out in a cleanroom for different deposition times and different concentrations of the AgNO_3_ solution to obtain various sizes and coverage ratios of flower-like nanostructures. The details of the liquid crystalline preparation and electrodeposition can be found in the Methods section.

### Morphology and constituent characterization

**Figure 1:**
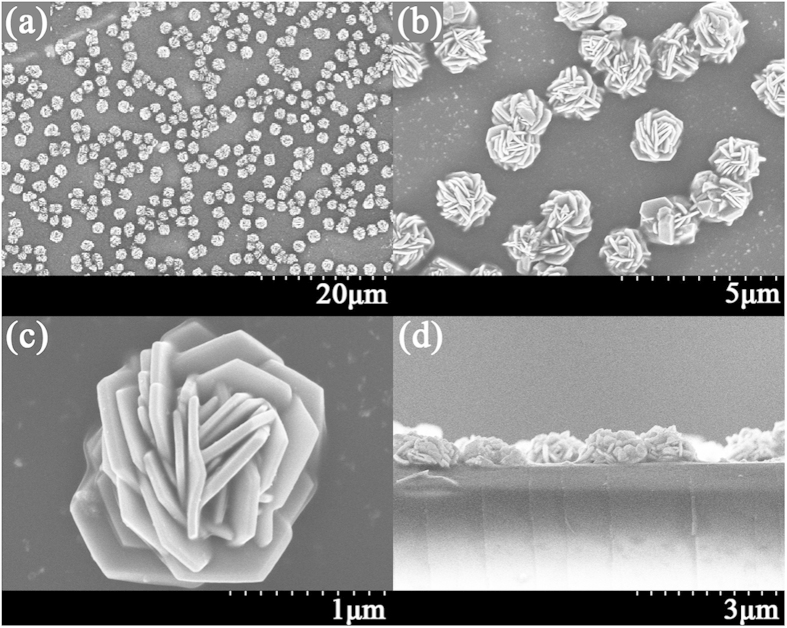
Scanning electron microscope images of the silver flower-like nanostructures. (**a**), (**b**) and (**c**) are top views of the silver flowers with different magnifications. (**d**) is a side view of the silver flowers. The growth time is 60 minutes, and the AgNO_3_ concentration is 0.1 mol/L.

Several electrodepositions for different growth times and AgNO_3_ concentrations are performed. Different sizes of flower-like silver nanostructures, ranging from 250 nm to 1.5 μm, are obtained. The field emission scanning electron microscope (FESEM) is used to characterize the size, shape and other morphological properties of the silver nanostructures. Figure 1 shows a FESEM image of the silver flower-like nanostructures that was obtained from the electrodeposition process.

**Figure 2:**
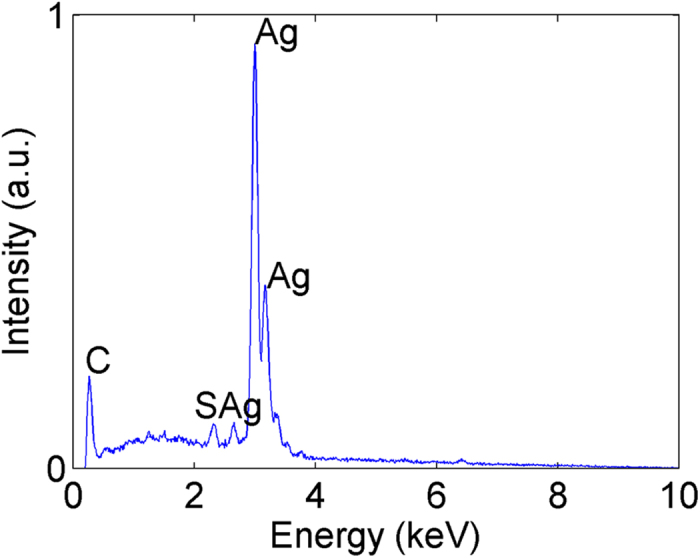
EDS spectrum of the silver flower-like nanostructures. Only peaks of silver, carbon and sulfur are observed. The peaks of carbon and sulfur can be attributed to the residual AOT in the sample. The flowers are composed primarily of silver.

In Fig. 1, we can observe the silver flowers. The AgNO_3_ concentration in the electrodeposition process is 0.1 mol/L and the growth time is 60 minutes. Figure 1(a) presents the FESEM image with low magnification and indicates that the electrodeposited silver nanostructures are quite uniform on the surface of the sample. In fact, the silver flowers are uniform over the entire working electrode in our experiment, and we believe that such uniform silver flowers can be achieved at an even larger scale. Figure 1(b) is a magnified image of (a) and shows that the sizes of each silver flower are also uniform. Different flowers have nearly the same size which indicates the same growth process for each flower. Figure 1(c) is the image of a single silver flower, where the morphology and size are clearly demonstrated. The size of the flower is approximately 1.5 μm. The shape is quite like a rose and is composed of a high density of petals. The thickness of the petals is approximately 25 nm to 100 nm. There are many horns on the petals and thin gaps between adjacent petals. Figure 1(d) is the side view of the silver flowers. The height is approximately 700 nm and is uniform for different flowers. From the image, we can also observe that there are no obvious differences in morphology (petal density, petal thickness, flower width) between the bottom and top of each flower. The elemental constitution of the silver flowers is analyzed using the energy-dispersive spectrum (EDS). The EDS pattern is shown in Fig. 2, from which only peaks of silver, carbon and sulfur are observed. The peaks of carbon and sulfur can be attributed to the residual AOT in the sample; because of the nanoscale space between the petals and flowers, the template molecule cannot be completely removed from the sample by the washing process. Thus, we believe that the flowers are composed primarily of silver.

### Growth mechanism and growth control

**Figure 3:**
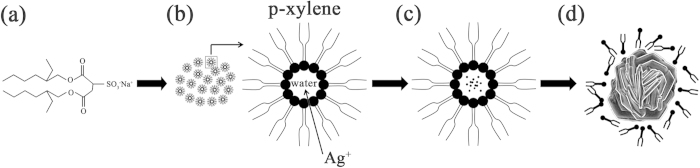
The schematic illustration for the growth process of flower-like silver nanostructures. (**a**) The molecular structure of AOT (C_20_H_37_NaO_7_S). The molecule contains a hydrophilic head and two hydrophobic chains. (**b**) The microphase-separated nanostructure of the liquid crystalline phase. (**c**) The initial silver dots electrodeposited at the beginning of the process. (**d**) The aggregated silver structures break the liquid crystalline phase and become flowerlike as the deposition process continues.

The growth mechanism of such flower-like nanostructures in the liquid crystalline phase can be attributed to the synergistic soft template mechanism^27^,^28^, which refers to the cooperative effect of the liquid crystalline phase soft template and the self-assembly of Ag. The AOT is a type of amphiphilic copolymer whose molecular structure contains a hydrophilic head and two hydrophobic chains, as Fig. 3(a) shows. When the AOT, p-xylene and water are mixed together, the hydrophilic heads of the amphiphilic copolymer aggregate to form a micelle with water inside and leave the hydrophobic tails outside in the p-xylene, which indicates that the process of microphase separation has started. When the phase equilibrium is achieved, the microphase-separated nanostructures in the liquid crystalline phase are many water pores surrounded by p-xylene^26^, as Fig. 3(b) shows. The Ag ions are encapsulated in the water pores. The initial nucleation of silver takes place in such pores only when deposition starts, as Fig. 3(c) illustrates. As the deposition process proceeds, the aggregated silver structures break the liquid crystalline phase template and become flowerlike due to the self-assembly effect of Ag, as shown in Fig. 3(d). The liquid crystalline phase acts as a soft template. The exact mechanism for the growth of such metal nanostructures in the liquid crystalline phase template remains a challenging research question. However, we can still use this method to fabricate such silver flower-like nanostructures conveniently.

**Figure 4:**
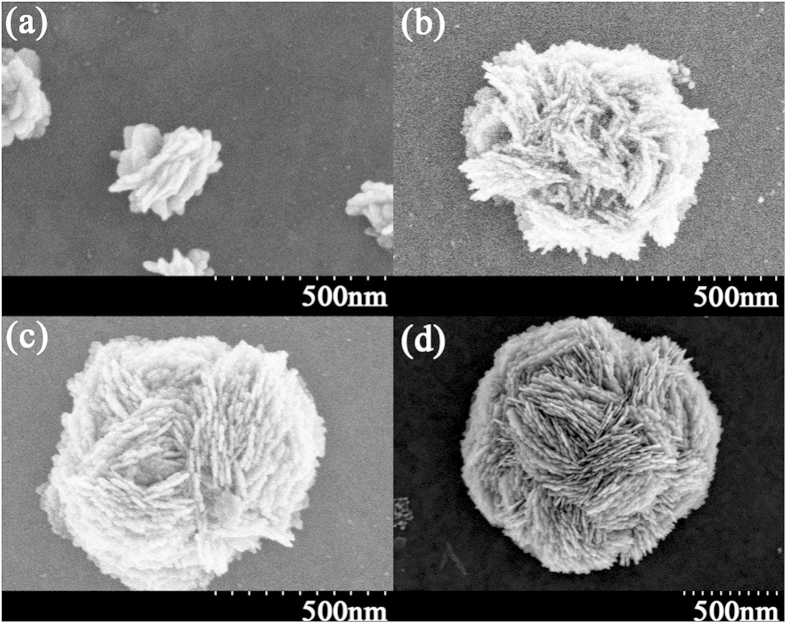
The scanning electron microscope images of different size flower-like silver nanostructures. (**a**) 250-nm-diameter flower-like nanostructures for 5 minutes of deposition time; (**b**) 600 nm for 10 minutes of deposition time; (**c**) 800 nm for 15 minutes of deposition time. (**d**) 1300 nm for 30 minutes. The AgNO_3_ concentration is 0.2 mol/L for the four images.

The size and morphology of the silver flower-like nanostructures depend primarily on the deposition time and the AgNO_3_ concentration. Figure 4 shows the FESEM images of silver flowers for different deposition times. From Fig. 4, we can observe different size flowers for different growth time. Figure 4(a) presents the image of silver flowers following 5 minutes of deposition time. The size of the flower is approximately 250 nm. Figure 4(b) shows the flowers after 10 minutes of deposition, and the size increases to approximately 600 nm. Figure 4(c) show the flowers for 15 and 30 minutes of deposition time, respectively. The sizes are approximately 800 nm and 1300 nm. From Fig. 4(d), we can observe that the petals of the flower are very dense and thin. The average petal thickness is approximately 10 nm, which indicates a large surface to mass ratio (up to 15 m^2^/g, roughly estimated). As Fig. 4 demonstrates, we can control the size of silver flowers by controlling the deposition time.

**Figure 5:**
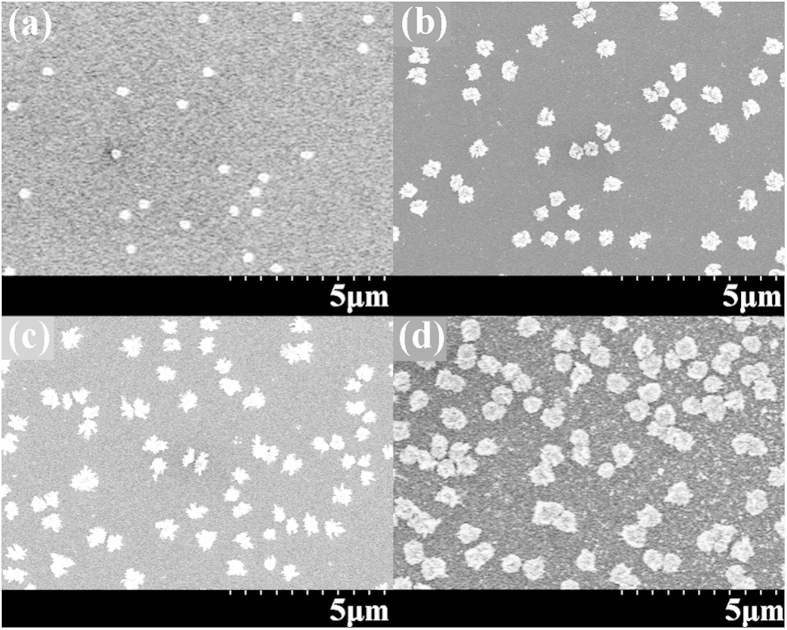
The scanning electron microscope images of different coverage ratio samples. (**a**) for 5 minutes of deposition time, and the coverage ratio is only approximately 1.8%; (**b**) for 10 minutes of deposition time, the coverage ratio is approximately 9.7%; (**c**) for 15 minutes of deposition time, the coverage ratio is approximately 16.3%. (**d**) for 30 minutes of deposition time, the coverage ratio is approximately 31.8%. The AgNO_3_ concentration is 0.1 mol/L for all samples.

**Figure 6:**
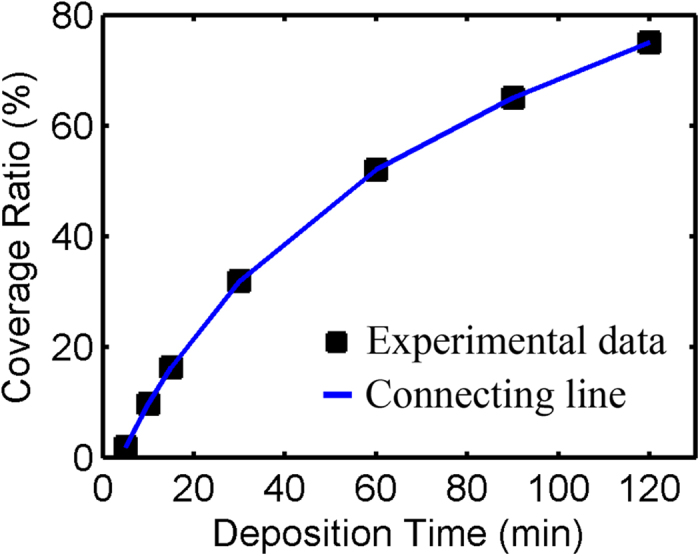
The relationship between silver flower coverage ratio and the deposition time. When the deposition time is less than 5 minutes, the coverage ratio is rather low. With the deposition time increasing, the coverage ratio increases continually, but the speed of increasing slows down noticeably.

To describe the density of the electrodeposited silver flowers, the coverage ratio is used, which refers to the ratio of the area covered by silver flowers to the total area of the working electrode. Figure 5 shows the FESEM images of different coverage ratio samples for different growth times. Figure 5(a) is an image for a 5-minute deposition, where the flowers are very small and sparse with a coverage ratio of only 1.8%. Figure 5(b) is for 10 minutes of deposition, and the coverage ratio is approximately 9.7%. Figure 5(c) are for 15 and 30 minutes, respectively, and the coverage ratios increase to 16.3% and 31.8%. The AgNO_3_ concentrations are all 0.1 mol/L for the four images in Fig. 5. Figure 6 illustrates the relationship between the coverage ratio and the deposition time. From Fig. 6, we can see that the coverage ratio of silver flowers increases with the deposition time. When the deposition time is less than 5 minutes, the silver nanostructures are small discrete dots instead of flowers, and the coverage ratio is rather low (not show in this work). As the deposition time increases, the coverage ratio increases continually, but the speed of increasing slows down noticeably.

**Figure 7:**
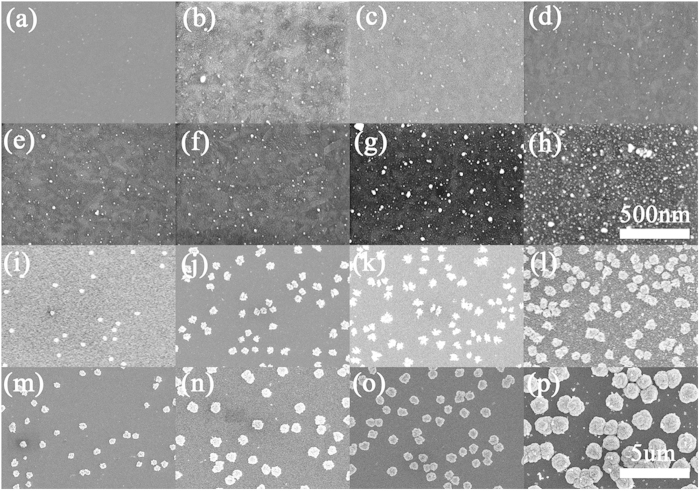
The overview FESEM images for different growth times and AgNO_3_ concentrations. (**a**)–(**d**) are images for the 0.001 mol/L AgNO_3_ concentration. (**e**)–(**h**) are for the 0.01 mol/L AgNO_3_ concentration. (**i**)–(**l**) are for the 0.1 mol/L AgNO_3_ concentration. (**m**)–(**p**) are for the 0.2 mol/L AgNO_3_ concentration. The four columns, from left to right, are for 5, 10, 15 and 30 minutes of growth time. The two scale bars are 500 nm for (**a**)–(**h**) and 5 μm for (**i**)–(**p**).

The AgNO_3_ concentration also plays an important role in the formation of the silver flower nanostructures. In this work, we also used other concentrations AgNO_3_ solution to perform electrodepositions. Figure 7 presents an overview of SEM images for different AgNO_3_ concentrations and growth times. The four rows from top to bottom are images for the 0.001, 0.01, 0.1 and 0.2 mol/L AgNO_3_ concentrations, respectively. The four columns from left to right are for 5, 10, 15 and 30 minutes of growth time. From Fig. 7(a)–(h), we can see that only small, discrete dots are obtained instead of flowers. That is, 0.001 and 0.01  mol/L concentrations are not sufficient for the formation of flowers in 30 minutes of growth time. For the 0.2 mol/L AgNO_3_ aqueous solution, the morphology of the silver nanostructures is different from those electrodeposited from the 0.1 mol/L AgNO_3_ aqueous solution. The flower petals are more dense and thin, as Fig. 4 shows, and the growth speed is faster, which can be observed in Fig. 7 (i)–(p). A high silver ion concentration is favourable for the formation of complicated structures. Generally, the increase of the silver ion concentration will also increase the growth speed of silver nanostructures during the electrodeposition process. Consequently, we can obtain more complicated flower-like silver structures quickly by increasing the AgNO_3_ concentration.

## Discussion

The silver flower-like nanostructures described above can be used as a substrate for SERS and SEF. SERS spectroscopy is a powerful and extremely sensitive spectroscopic tool that can provide a spectral fingerprint of a molecule. Generally, two different mechanisms are thought to cause the SERS phenomenon. One is the enhancement of local electromagnetic fields due to the surface plasmon resonance in metals, and the other is the chemical enhancement that originates from the charge transfer between the molecule and the metal surface. The surface plasmon resonance is generally believed to be the main contributor to SERS and to be several orders stronger than the charge transfer. Because the silver flowers described above have so many horns and thin gaps between the flowers and petals, we believe that a strong Raman enhancement effect will be achieved. Such nanostructures also can be used as a surface-enhanced fluorescence substrate. The enhancement mechanism of SEF is also understood in terms of hot spots, which are related to the excitation of the surface plasmon. The existing studies have shown that the localized field can achieve large fluorescence enhancements by factors ranging up to a few hundred^13^. This material may also have applications in chemical catalysis because of its large surface to mass ratio. Studies concerning SERS and SEF are still ongoing in our group; the main focus of this paper is fabrication. The novel fabrication method, which is very simple, inexpensive and suitable for large-scale fabrications, will make it easier to apply such flower-like silver nanostructures as SERS and SEF substrates.

In summary, we have demonstrated a novel method for the large-scale uniform fabrication of flower-like silver nanostructures. This novel fabrication method, which is quite simple and inexpensive, performs electrodeposition primarily in a ternary liquid crystalline phase. Different sizes and different coverage ratios of silver flowers are obtained by controlling the growth time and the concentration of silver nitrate. These silver flower nanostructures, which possess properties such as hot spots and large surface-to-mass ratios, can serve as surface-enhanced Raman scattering and surface-enhanced fluorescence substrates and may also have applications in photoluminescence and catalysis.

## Methods

### Materials and preparation of liquid crystalline phase

In this work, we use the liquid crystalline phase as a soft template to perform the electrodeposition process. The liquid crystalline phase is prepared according to the ternary phase diagram^26^, which consists of the anionic surfactant sodium bis(2-ethylhexyl) sulfosuccinate (AOT), the oil phase p-xylene, and water(AgNO_3_ aqueous solution in this work). The purities of AOT, p-xylene and silver nitrate are 98.0 wt%, 99.0 wt% and 99.9 wt%, respectively. All chemical materials are used directly from the manufacturer without further purification. Deionized water with 18 MΩ·cm resistivity is used as a solvent. In our experiment, we use an aqueous AgNO_3_ solution instead of water. The concentrations of the AgNO_3_ solutions are 0.001, 0.01, 0.1 and 0.2 mol/L, respectively. The AOT is dissolved in the p-xylene to obtain a solution with 1.4 mol/L concentration. The AOT solution and AgNO_3_ solution are mixed together with a [H_2_O]/[AOT] molar ratio of 10. After 1 hour of vigorous stirring, the mixture becomes to a clear homogeneous yellow liquid. Due to the silver ions in the liquid, the entire stirring process is performed in a black glass bottle, and the temperature of the laboratory is approximately 20 °C.

### Electrodeposition of flower-like silver nanostructures

To keep the surface clean, the electrodeposition process is prepared and carried out in a cleanroom. The liquid crystalline phase is used as the electrolyte. The anode is a one-millimetre-thick polished silver plate. Indium tin oxide glass (ITO glass) is used as the cathode, which is also the working electrode to collect the flower-like silver nanostructures. The size of both electrodes is 20 mm × 20 mm. Before electrodeposition, the electrodes are cleaned completely with ethanol and deionized water. These procedures can guarantee that only the silver dissolution reaction occurs on the anode and that the liquid crystalline phase is not disturbed during the electrodeposition process. The 2.0 V static potential is applied to the electrodes by the DF1761 potentiostat. The distance between cathode and anode is approximately 0.5 mm, which is determined by the spacers between them. The current density increased gradually from approximately 10 μA/cm^2^ to 120 μA/cm^2^ with the deposition time due to the increase of the surface area. As the growth process goes on, more and more flowers appear, the size of flowers increases and the morphology becomes more complicated. In this work, we perform the deposition process for 5, 10, 15, 30, and 60 minutes to obtain different sizes and coverage ratios for the flower-like nanostructures. After deposition, the working electrode with silver flower-like nanostructures is washed by ethanol and deionized water to remove the liquid crystalline phase left on the electrode.

## Additional Information

**How to cite this article**: Yang, C. *et al.* Large-scale controlled fabrication of highly roughened flower-like silver nanostructures in liquid crystalline phase. *Sci. Rep.* **5**, 12355; doi: 10.1038/srep12355 (2015).

## References

[b1] Zhang F. *et al.* Fabrication of Ag@SiO_2_@Y_2_O_3_: Er nanostructures for bioimaging: tuning of the upconversion fluorescence with silver nanoparticles. J. Am. Chem. Soc. 132, 2850–2851 (2010). 20158187 10.1021/ja909108x

[b2] Li Y., Wu Y. & Ong B. S. Facile synthesis of silver nanoparticles useful for fabrication of high-conduvtivity elements for printed electronics. J. Am. Chem. Soc. 127, 3266–3267 (2005). 15755129 10.1021/ja043425k

[b3] Gude K. & Narayanan R. Synthesis and characterization of colloidal supported metal nanoparticles as potential intermediate nanocatalysts. J. Phys. Chem. Soc. 114, 6356–6362 (2010).

[b4] Li Y. & Shi G. Electrochemical growth of two-dimensional gold nanostructures on a thin polypyrrole file modified ITO electrode. J. Phys. Chem. B. 109, 23787–23793 (2005). 16375362 10.1021/jp055256b

[b5] He Y., Yu X. & Yang B. Novel Cu flower-like nanostructures synthesized from a solid-stabilized emulsion approach. Mater. Chem. Phys. 99, 295–299 (2006).

[b6] Jin R. *et al.* Photoinduced conversion of silver nanospheres to nanoprisms. Science 294, 1901–1903 (2001). 11729310 10.1126/science.1066541

[b7] Gould I. R., Lenhard J. R., Muenter A. A., Godleski S. A. & Farid S. Two-electron sensitization: a new concept for silver halide photography. J. Am. Chem. Soc. 122, 11934–11943 (2000).

[b8] Challener W. A., Ollmann R. R. & Kam K. K. A surface Plasmon resonance gas sensor in a ‘compact disc’ format. Sensors Actuators B. 56, 254–258 (1999).

[b9] Shanmukh S. *et al.* Rapid and sensitive detection of respiratory virus molecular signatures using a silver nanorod array SERS substrate. Nano Lett. 6, 2630–2636 (2006). 17090104 10.1021/nl061666f

[b10] Liu G. & Lee L. P. Nanowell surface enhanced Raman scattering arrays fabricated by soft-lithography for label-free biomolecular detections in integrated microfluidics. Appl. Phys. Lett. 87, 074101 (2005).

[b11] Alvarez-Puebla R., Cui B., Bravo-Vasquez J., Veres T. & Fenniri H. Nanoimprinted SERS-active substrates with tunable surface plasmon resonances. J. Phys. Chem. C. 111, 6720–6723 (2007).

[b12] Tian Z., Ren B., Li J. & Yang Z. Expanding generality of surface-enhanced Raman spectroscopy with borrowing SERS activity strategy. Chem Commun. 34, 3514–3534 (2007). 10.1039/b616986d 18080535

[b13] Dong J., Zheng H., Yan X., Sun Y. & Zhang Z. Fabrication of flower-like silver nanostructure on the AI substrate for surface enhanced fluorescence. Appl. Phys. Lett. 100, 051112 (2012).

[b14] Bratlie K. M., Lee H., Komvopoilos K., Yang P. & Somorjai G. A. Platinum Nanoparticle shape effects on benzene hydrogenation selectivity. Nano Lett. 7, 3097–3101 (2007). 17877408 10.1021/nl0716000

[b15] Horiuchi Y., Shimada M., Kamegawa T., Mori K. & Yamashita H. Size-controlled synthesis of silver nanoparticles on Ti-containing mesoporous silica thin film and photoluminescence enhancement of rhodamine 6G dyes by surface plasmon resonance. J. Mater. Chem. 19, 6745–6749 (2009).

[b16] Atwater H. A. & Polman A. Plasmonics for improved photovoltaic devices. Nat. Mater. 9, 205–213 (2010). 20168344 10.1038/nmat2629

[b17] Witten T. A. & Sander L. M. Diffusion-limited aggregation, a kinetic critical phenomenon. Phys. Rev. Lett. 47, 1400–1403 (1981).

[b18] Ben-Jacob E. & Garik P. The formation of patterns in non-equilibrium growth. Nature, 343, 523–530 (1990).

[b19] Kolb M., Botet R. & Jullien R. Scaling of kinetically growing clusters. Phys. Rev. Lett. 51, 1123–1126 (1983).

[b20] Yang J., Dennis R. C. & Sardar D. K. Room-temperature synthesis of flowerlike Ag nanostructures consisting of single crystalline Ag nanoplates. Mater. Res. Bull. 46, 1080–1084 (2011).

[b21] Hong L., Li Q., Lin H. & Li Y. Synthesis of flower-like silver nanoarchitectures at room temperature. Mater. Res. Bull. 44, 1201–1204 (2009).

[b22] Sackmann M., Bom S., Balster T. & Materny A. Nanostructured gold surfaces as reproducible substrates for surface-enhanced Raman spectroscopy. J. Raman Spectrosc. 38, 277–282 (2007).

[b23] Kumar P. S., Pastoriza-Santos I., Rpdriguez-Gonzalez B., Garcia de Abajo F. J. & Liz-Marzan L. M. High-yield synthesis and optical response of gold nanostars. Nanotechnolog 19, 01506 (2008). 10.1088/0957-4484/19/01/015606 21730541

[b24] Whitney A. V., Elam J. W., Stair P. C. & Van Duyne R. P. Toward a Thermally Robust operando surface-enhanced raman spectroscopy substrate. J.Phys. Chem. C 111, 16827–16832 (2007).

[b25] Mohanty A., Garg N. & Jin R. A universal approach to the synthesis of noble metal nanodendrites and their catalytic properties. Angew. Chem. Int. Ed. 49, 4962–4966 (2010). 10.1002/anie.201000902 20540128

[b26] Ekwall P., Mandell L. & Fontell K. Solubilization in micelles and mesophases and the transition from normal to reversed structures. Mol. Cryst. Liquid Cryst. 8, 157–213 (1969).

[b27] Wu W. *et al.* *In situ* formation of Ag flowerlike and dendritic nanostructures in aqueous solution and hydrolysis of an amphiphilic block copolymer. Nanotechnology 16, 2048–2051 (2005). 20817969 10.1088/0957-4484/16/10/011

[b28] Wang Y. & Zhao D. Y. On the controllable soft-templating approach to mesoporous silicates. Chem. Rev. 107, 2821–2860 (2006). 10.1021/cr068020s 17580976

